# Sarcopenia and sepsis fuel a self-perpetuating cycle of immunometabolism decline

**DOI:** 10.3389/fimmu.2026.1867909

**Published:** 2026-07-21

**Authors:** Elisa Gouvea Gutman, Renan Muniz-Santos, Adilson Moreira da Silva, Giovanna Lucieri Alonso Costa, Carolina Medina Coeli da Cunha, Bianca Portugal Tavares de Moraes, Cassiano Felippe Gonçalves−de−Albuquerque

**Affiliations:** 1 Translational Neuroscience Laboratory (LabNet), Biomedical Institute, Federal University of the State of Rio de Janeiro, Rio de Janeiro, Brazil; 2 Clinical Medicine Graduate Program, Federal University of Rio de Janeiro, Rio de Janeiro, Brazil; 3 Lorraine Protein Biochemistry Group, Graduate Program in Neurology, Gaffrée e Guinle University Hospital, Rio de Janeiro, Brazil; 4 Immunopharmacology Laboratory and Postgraduate Degree in Molecular and Cellular Biology, Federal University of State of Rio de Janeiro, Rio de Janeiro, Brazil; 5 Institute of Scientific, Technological, and Communication Information (ICICT), Oswaldo Cruz Institut (FIOCRUZ), Rio de Janeiro, Brazil; 6 Immunopharmacology Laboratory, Gradute Program in Molecular and Cell Biology, Oswaldo Cruz Institut (FIOCRUZ), Rio de Janeiro, Brazil

**Keywords:** immunometabolism, muscle-immune crosstalk, myokines, sarcopenia, sepsis, skeletal muscle

## Abstract

Sepsis and sarcopenia are intertwined clinical challenges characterized by profound immunometabolic dysregulation. Traditionally viewed as a passive reservoir, skeletal muscle is now recognized as an active immunometabolic rheostat that dynamically influences systemic inflammation and homeostasis. This review synthesizes recent advances that redefine our understanding of muscle in critical illness, moving beyond simplistic views of catabolism to encompass complex adaptive strategies. This review summarizes recent advances and discusses septic autocannibalism, a process in which muscle proteolysis, driven by a metabolic defense priority, provides key substrates, glutamine for immune function and alanine for hepatic gluconeogenesis. Initially adaptive, sustained activation of this response leads to severe muscle wasting and long-term functional impairment. We analyze the bidirectional relationship between these conditions, focusing on shared risk factors such as immunosenescence and obesity. Key molecular pathways, including the IL-6/JAK/STAT and NF-κB axes, are examined, with particular emphasis on how the temporal release of myokines (e.g., IL-6, IL-15, IGF-1) dictates their shift from adaptive signals to chronic catabolic drivers. Furthermore, we discuss how energy reprogramming, characterized by aerobic glycolysis and mitochondrial failure, disrupts muscle homeostasis. We highlight physical activity as a potent modulator of immunometabolic health through the release of anti-inflammatory myokines and enhanced mitochondrial biogenesis. To propel the field forward, we propose experimental avenues: single-cell spatial transcriptomics to map cellular crosstalk, mitochondrial transplantation to restore energetic capacity, and the identification of “muscle resilience” biomarkers for early intervention. This review underscores the urgent need for integrated immunometabolic approaches to improve outcomes in critically ill patients.

## 1 Introduction

Sarcopenia is a progressive skeletal muscle disorder characterized by loss of muscle strength, reduced muscle mass, and impaired physical performance (1). Although traditionally defined in the context of aging, it is increasingly recognized to occur in younger individuals as well (1, 2). It is also a significant public health issue that may result in various adverse outcomes, such as falls, disability, frailty, and reduced quality of life (3). It is considered a predictor of all-cause mortality among hospitalized patients (4). Given that emerging evidence suggests an immunoregulatory role for skeletal muscle, the potential impact of sarcopenia on inflammatory diseases has been increasingly studied, particularly sepsis, a condition characterized by a dysregulated immunometabolic response to infection. For instance, a meta-analysis showed that septic patients with sarcopenia had a significantly increased risk of acute and post-discharge mortality (5). Also, the features of sarcopenia, such as reduced muscle mass and impaired physical function, combined with their impact on immune response, can support sepsis development or progression (6–8).

Conversely, sarcopenia may develop as a consequence of hypermetabolic and inflammatory states, such as sepsis. Septic environments are associated with the release of pro-inflammatory cytokines, activation of the sympathetic nervous system (including the use of intravenous catecholamines), limited nutritional intake, and physical inactivity, leading to muscle loss due to increased catabolism and decreased anabolic signaling (9). This profound metabolic dysregulation, particularly the accelerated depletion of amino acids from skeletal muscle, is often conceptualized as septic autocannibalism (10), a critical host response aimed at supplying essential substrates for immune function and gluconeogenesis during severe infection.

Both sepsis and sarcopenia can culminate in a considerable impact on patient morbidity and mortality across the lifespan. Although traditionally investigated as separate entities - sepsis as an acute dysregulated inflammatory response to infection, and sarcopenia as a chronic, often age-related, loss of muscle mass - their interplay has now been acknowledged (11). Indeed, the relationship between these conditions can be bidirectional and is rooted in shared biological, modifiable, and environmental risk factors that may accumulate over time. For instance, advanced age, physical inactivity, chronic disease, obesity, and smoking are interconnected factors that can also drive each other, creating vicious cycles that can trigger and aggravate systemic inflammation and metabolic dysregulation (12, 13).

Beyond shared risk factors, both conditions can create, at the cellular and molecular levels, an immunometabolic background that exacerbates the other’s effects. The immune-modulatory roles of skeletal muscle-derived molecules - such as myokines and heat shock proteins (HSPs) - highlight the crucial integration of muscle tissue into the systemic immune response (14, 15). Moreover, the hypermetabolic state of sepsis impacts energy metabolism pathways. It is characterized by energy inefficiency, mitochondrial failure, oxidative stress, and mobilization of energy supplies to tissues. Beyond the catabolic muscle loss already described, these immunometabolic changes further hinder muscle regeneration, creating a harmful feedback loop that can exacerbate sarcopenia (16).

Recent conceptual advances have reshaped the understanding of the sepsis-sarcopenia axis from a model in which skeletal muscle was viewed merely as a passive target of sepsis-induced catabolism into one in which it is an active node of a dynamic immunometabolic circuit. Emerging evidence suggests that skeletal muscle acts not only as a target of catabolism but as an active regulator of systemic immune responses through myokine signaling, mitochondrial-derived danger signals, and metabolic substrate redistribution (17). Notably, recent studies highlight mitochondrial quality control failure and inflammasome activation as convergent nodes linking muscle wasting to immune dysfunction (18–20). These findings challenge the classical view of muscle as a passive reservoir and instead position it as a central immunometabolic organ in the pathophysiology of sepsis (16).

In this review, we propose that the interaction between sarcopenia and sepsis can be conceptualized as a unified immunometabolic syndrome driven by reciprocal feedback between skeletal muscle and the immune system. We synthesize recent findings on shared risk factors, molecular signaling pathways, and metabolic reprogramming, with particular emphasis on mechanisms that have emerged in recent years. Furthermore, we highlight key areas of controversy, identify unresolved mechanistic questions, and propose experimental and translational directions to disrupt this pathological cycle. By integrating insights from muscle biology, immunology, and metabolism, this work aims to provide a framework for understanding and ultimately targeting the complex interplay between sarcopenia and sepsis.

### 1.1 Scope and literature search

This work is a narrative review aimed at summarizing current knowledge regarding the interplay between sarcopenia, sepsis, and immunometabolic dysfunction. Relevant literature was identified through searches of the PubMed/MEDLINE, Scopus, and Web of Science databases conducted between January and March 2026. The search strategy combined the terms “sarcopenia”, “sepsis”, “skeletal muscle”, “muscle wasting”, “myokines”, “immunometabolism”, “inflammaging”, “inflammation”, “mitochondrial dysfunction”, “exercise”, “mitochondrial dysfunction”, “obesity”, “visceral obesity”, “aging”, “chronic low-grade inflammation”, and “amino acid metabolism”, using Boolean operators when appropriate.

No strict date restriction was applied; however, emphasis was placed on studies published within the last 10 years to capture recent advances in immunometabolism and skeletal muscle biology. Earlier landmark studies were included when they provided foundational concepts or mechanistic insights relevant to the topic. Priority was given to original human studies, systematic reviews, meta-analyses, and mechanistic preclinical investigations that directly addressed the relationship between sarcopenia, sepsis, skeletal muscle function, inflammation, and metabolic regulation. Additional references were identified through manual screening of reference lists from key articles. Because this is a narrative review, article selection was based on scientific relevance and conceptual contribution rather than a predefined systematic review protocol, which represents an inherent limitation of the study.

## 2 Shared risk factors for sarcopenia and sepsis: behavioral and disease-related factors

**Figure 1:**
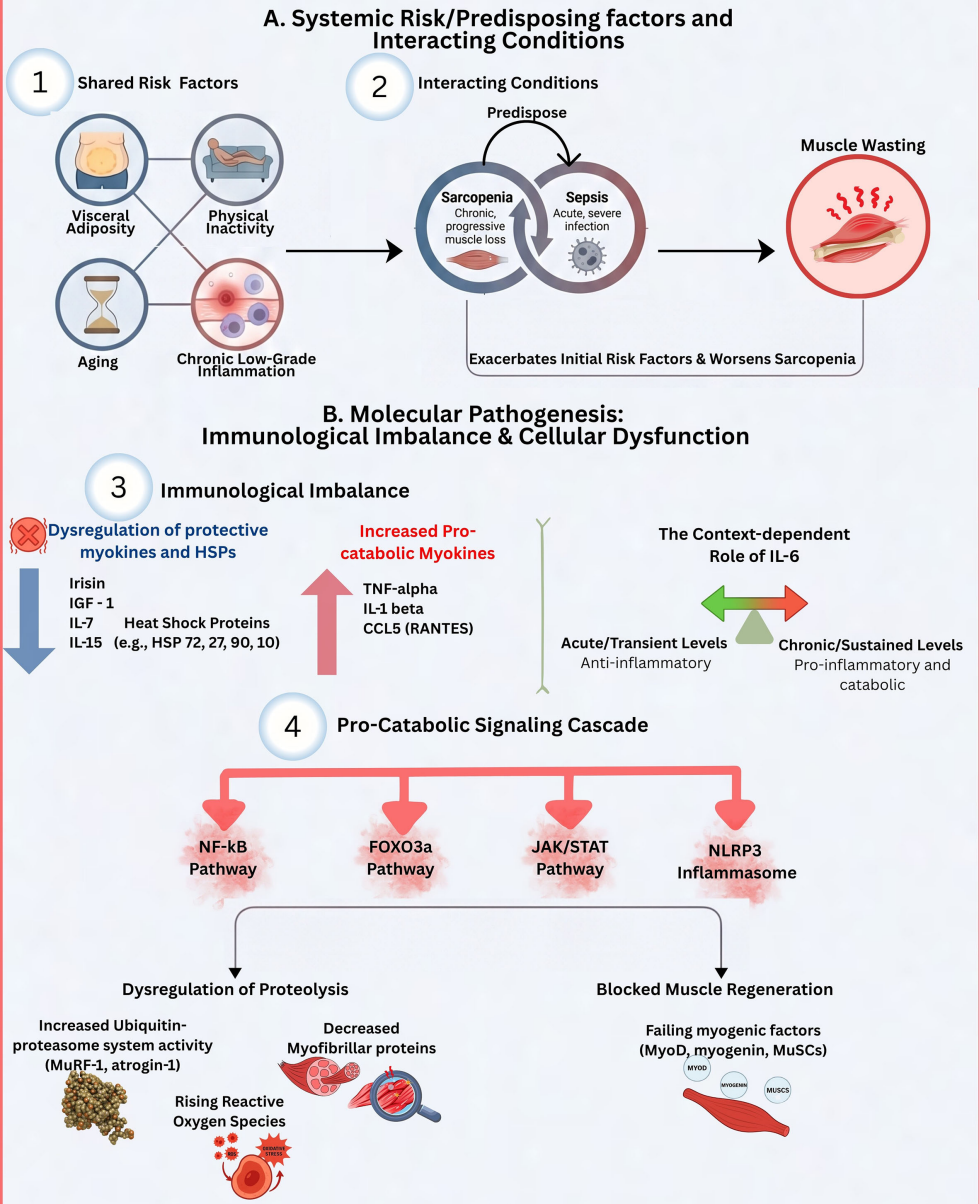
Pathophysiological framework linking sarcopenia and sepsis: from systemic risk factors to molecular muscle injury. **(A)** Systemic risk/predisposing factors and interacting conditions. Shared, largely modifiable risk factors - visceral adiposity, physical inactivity, aging, and chronic low-grade inflammation - establish a baseline of host vulnerability that predisposes to the two interacting conditions, sarcopenia (chronic, progressive muscle loss) and sepsis (acute, severe infection). Their interaction culminates in muscle wasting, which in turn exacerbates the initial risk factors and further worsens sarcopenia, sustaining a self-perpetuating cycle. **(B)** Molecular pathogenesis: immunological imbalance and cellular dysfunction. The interaction produces an immunological imbalance characterized by downregulation of protective myokines (irisin, IGF-1, IL-7, IL-15) and heat shock proteins (HSP72, HSP27, HSP90, HSP10), along with increased pro-catabolic myokines (TNF-α, IL-1β, CCL5/RANTES). IL-6 is depicted as a context-dependent mediator: anti-inflammatory at acute, transient concentrations but pro-inflammatory and catabolic when chronically sustained. These signals converge on a pro-catabolic signalling cascade (NF-κB, FOXO3a, JAK/STAT, and the NLRP3 inflammasome) driving two effector programmes: dysregulated proteolysis — increased ubiquitin–proteasome system activity (E3 ligases MuRF-1 and atrogin-1), declining myofibrillar protein content, and rising reactive oxygen species (ROS) — and blocked muscle regeneration through failing myogenic factors [MyoD, myogenin, and muscle satellite cells (MuSCs)]. CCL5, C–C motif chemokine ligand 5; HSP, heat shock protein; IGF-1, insulin-like growth factor 1; IL, interleukin; MuSCs, muscle satellite cells; MyoD, myoblast determination protein 1; NF-κB, nuclear factor kappa B; NLRP3, NLR family pyrin domain containing 3; ROS, reactive oxygen species; TNF-α, tumour necrosis factor alpha.

There is growing recognition of the importance of identifying and addressing risk factors for sepsis and sarcopenia in a timely, targeted manner. The shared biological hazards that predispose individuals to both sepsis and sarcopenia may accumulate years before the clinical onset of either condition. Throughout this review, these chronic processes - including inflammaging, chronic low-grade inflammation, obesity, frailty, and baseline sarcopenia - are considered primarily as pre-existing host vulnerabilities that heighten susceptibility to severe infection and predispose to worse sepsis outcomes and impaired recovery, rather than as features of the acute septic response itself. The first link between sepsis and sarcopenia stems from potential shared factors underlying these conditions. There are distinct elements that can impact the individual risk of developing sarcopenia and sepsis, including lifestyle and disease-related factors, such as advanced age, physical inactivity, chronic disease, malnutrition, smoking, and obesity (**Figure 1**). These risk factors are interconnected, meaning one factor can increase the risk of another. Some conditions related to sarcopenia and sepsis are described below.

### 2.1 Chronic low-grade inflammation

CLGI is a pivotal pre-existing condition that shapes susceptibility to, and the severity of, both sepsis and sarcopenia (21, 22). For instance, CLGI serves as an intersection point among aging, obesity, chronic diseases (e.g., diabetes), and the immunological response to infections, as it may induce or be induced by these conditions, creating a snowball effect (positive feedback loop) (23). It is also associated with muscle wasting and impaired skeletal muscle-brain crosstalk, through insulin resistance to mitochondrial dysfunction (24).

The muscle-immune system connection is bidirectional: CLGI induces muscle catabolism through inflammatory pathways, leading to insufficient myokine signaling, alterations in membrane-bound factors toward a pro-inflammatory profile, and impaired regenerative capacity in immune cells. This disruption of immune function in muscle tissue perpetuates CLGI (25). Withal, CLGI may hinder the stimulatory effects of protein ingestion on protein synthesis and the inhibitory effect on proteolysis.

### 2.2 Physical inactivity

**Figure 2:**
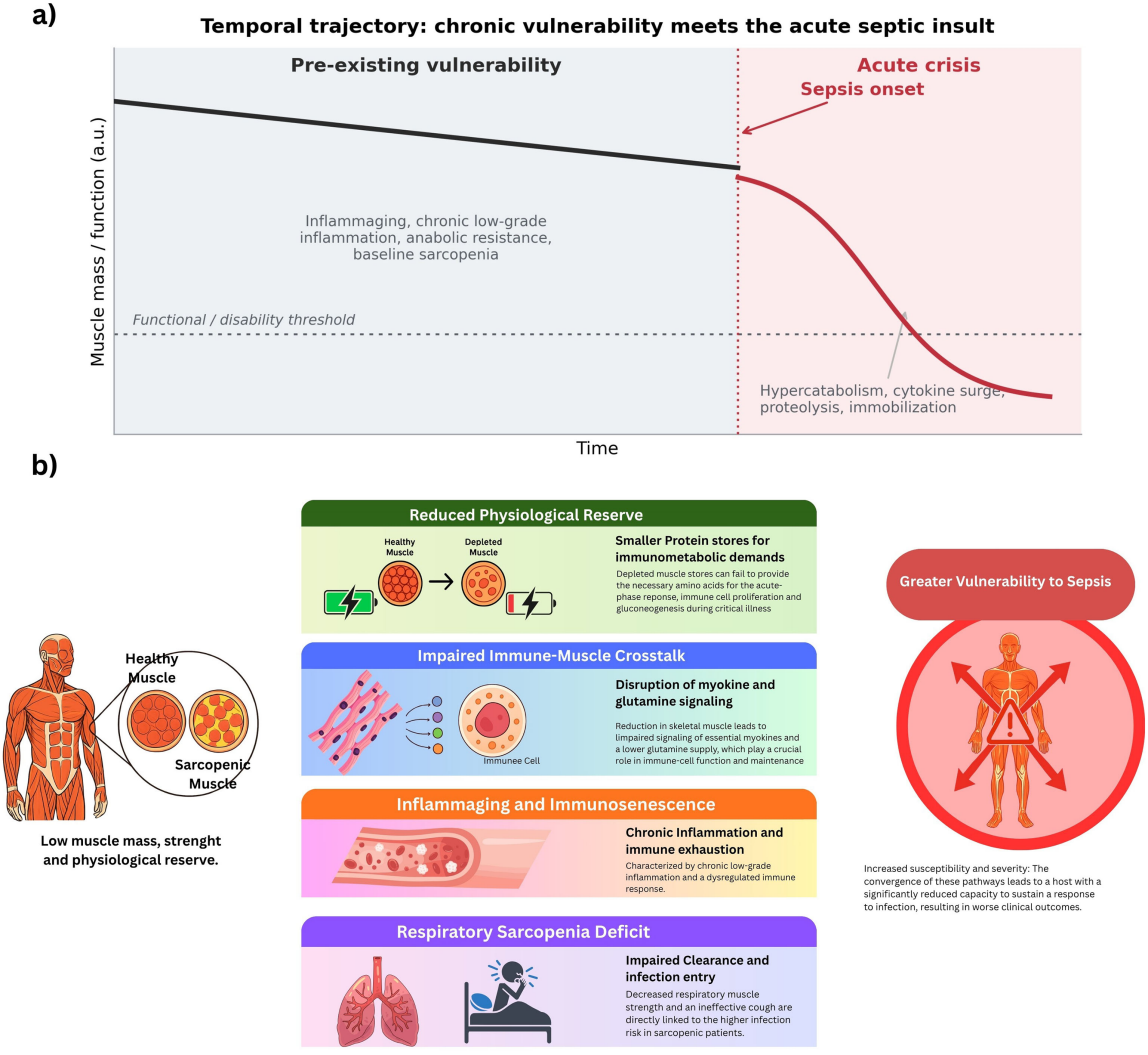
Sarcopenia as a pre-existing vulnerability that predisposes to sepsis. **(A)** Temporal trajectory. Skeletal muscle mass and function are shown over time theoretically. During a pre-existing sarcopenic (chronic) phase, inflammaging, chronic low-grade inflammation, anabolic resistance, and baseline sarcopenia drive a slow decline that establishes host vulnerability; at sepsis onset, an acute catabolic crisis can produce a steep fall in muscle mass and function, frequently crossing the functional/disability threshold. **(B)** Mechanisms of predisposition. Low muscle mass and reduced physiological reserve increase vulnerability through four complementary mechanisms: reduced physiological reserve (diminished protein/amino-acid stores for the acute-phase, immune, and gluconeogenic demands of critical illness); impaired immune–muscle crosstalk (reduced myokine and glutamine signaling supporting immune-cell function and maintenance); inflammaging and immunosenescence (chronic low-grade inflammation with an exhausted, dysregulated immune response); and respiratory sarcopenia (reduced respiratory muscle strength and ineffective cough impairing airway clearance and easing pathogen entry). These pathways converge on greater vulnerability to sepsis, increasing both susceptibility to infection and the severity of subsequent disease. a.u., arbitrary units.

Physical inactivity has emerged as a particularly significant risk factor, with a large body of evidence linking sedentary behavior with an increased risk of sarcopenia across multiple populations. Conversely, sarcopenia can lead to impaired mobility and reduced physical activity, increasing the risk of infections and sepsis (26, 27), which can then further exacerbate muscle wasting and loss of function (**Figure 2A**).

Regular exercise practice is identified as a critical preventive and therapeutic intervention for sarcopenia, improving skeletal muscle metabolism and function. For example, former Olympic athletes have been shown to have a lower incidence of sarcopenia, particularly among those who continued to exercise and participated in more intense sports (28). Nonetheless, little physical activity may contribute to malnutrition, which exacerbate the poor functional status of patients with sarcopenia and/or sepsis (29).

Physical activity is a key factor in maintaining immune function in elderly patients, with increased numbers of naïve T-cells, improved neutrophil function, and enhanced NK-cell cytotoxicity, thereby improving vaccine responses and reducing CLGI (30). Thus, exercise may protect immune and muscle function against the detrimental effects of aging. As physical activity is inversely correlated with all-cause mortality, exercise is emerging as a substantial contributor to longevity (31). Despite the practical challenges of implementation, exercise may be critical to protecting an aging population against the clinical consequences of sarcopenia and immune senescence, thereby reducing the risk of sepsis.

Beyond reducing ambulatory activity, sarcopenia also compromises the respiratory musculature, the so-called “respiratory sarcopenia” (32) (**Figure 2B**). This triggers a dangerous downward spiral, in which poor respiratory capacity leads to further declines in physical activity and worsening fatigue, thereby accelerating muscle wasting (33, 34). Moreover, the age- and disease-related loss of mass and strength in the diaphragm, intercostal, and accessory respiratory muscles reduces maximal inspiratory and expiratory pressures and weakens cough (35–37). This, in turn, impairs airway clearance and secretion handling, favoring the retention of pathogens in the airways and predisposing to infections such as pneumonia (38). Given that the respiratory tract is among the most frequent sources of sepsis, respiratory sarcopenia provides a mechanical route by which a decreased pre-existing muscle reserve facilitates pathogen entry and may increase the risk of sepsis (39) (**Figure 2B**).

### 2.3 Aging

Aging is often accompanied by changes in body composition that lead to a shift toward decreased muscle mass and increased fat mass, even in relatively weight-stable, healthy individuals. Aging is a risk factor for various clinical conditions in humans, including sepsis and sarcopenia. Sarcopenia has long been recognized as a geriatric syndrome (although it can also occur in younger people), and sepsis prevalence is much higher in the elderly (7, 40).

The immunosenescence observed in elderly patients involves functional impairment of both cell-mediated immunity and humoral immunity, resulting in a poor host response to infections. This not only increases the risk of sarcopenia and sepsis but also contributes to a more severe presentation and higher mortality rates (29). A senescence-associated secretory phenotype (SASP) is commonly observed in senescent cells resident in skeletal muscle that accumulate with age, characterized by the overexpression of pro-inflammatory mediators such as Interleukin-1β (IL-1β), IL-6, IL-8, tumor necrosis factor-α (TNF-α), and Interferon-γ (IFN-γ), thereby promoting a pro-inflammatory environment (41). The inflammation associated with aging is known as inflammaging and can result from the aging process itself or due to an increased likelihood of underlying comorbidities. Increased serum levels of C-reactive Protein (CRP), a well-studied biomarker of inflammation and sepsis, are associated with age-related sarcopenia (42).

Along these lines, the aging process is intricately linked to heightened CLGI, as it alters the intestinal microbiota, promoting malnutrition, exacerbating inflammation, and increasing susceptibility to infection, thereby contributing to the pathophysiologic processes of sarcopenia and sepsis (43, 44). The aging process is also associated with elevated production of reactive oxygen species (ROS) and imbalances in hormone secretion. Consequently, the diminished presence of anabolic hormones, such as growth hormone and steroids, can contribute to reduced muscle growth and maintenance (45). Other factors associated with aging, such as neuronal changes, poor physical activity, anorexia, or malnutrition, also contribute to an increased risk of sepsis and sarcopenia (29, 42). Hence, these alterations combined increase the risk of sarcopenia and sepsis development.

### 2.4 Visceral obesity

The accumulation of excess intra-abdominal adipose tissue characterizes visceral obesity. It is part of a phenotype that includes dysfunctional expansion of subcutaneous adipose tissue and ectopic triglyceride storage and is a pre-existing condition closely linked to worse outcomes in metabolic and inflammatory diseases, such as sepsis and sarcopenia. Sarcopenia, sepsis, and visceral obesity may represent interconnected disorders that act as independent risk factors for the aggravation of each other and as mutual stimulants to precipitate their health-threatening effects synergistically, as demonstrated in the multivariate analysis of an observational study with 236 surgical ICU patients (46).

Emerging evidence suggests that adipose tissue undergoes significant reprogramming during sepsis, shifting from a passive energy reservoir to an active immunometabolic hub. This functional shift may be driven by a metabolic defense priority, where the preferential mobilization of fat aims to spare skeletal muscle protein, a strategy that, if compromised, can exacerbate sarcopenia (47).

Several feedback mechanisms and overlapping factors have been implicated in the crosstalk between these diseases. For example, sarcopenia is associated with leptin resistance and hyperleptinemia, which in turn lead to insulin resistance (48). It can also reduce energy expenditure, contributing to weight gain (49). On the other hand, increased intramuscular fat infiltration may engender metaflammation, a specific type of chronic inflammation caused by nutrient excess, which can induce the secretion of pro-inflammatory cytokines and adipokines, such as leptin, generating obesity-related insulin resistance and muscle atrophy (50). Moreover, excessive lipids in the form of free fatty acids (FFAs) induce mitochondrial dysfunction, disrupt β-oxidation of FFAs, and enhance ROS production, resulting in dysregulated autophagy and apoptosis of muscle cells, lipotoxicity, and impaired myokine release, as seen in sarcopenia (51, 52).

Regarding sepsis, the role of obesity remains controversial. Piling evidence has highlighted the “obesity paradox” in sepsis, which is defined as the finding that obese people may have better outcomes than their normal weight or underweight counterparts (53). Nonetheless, these findings should be interpreted with caution because the survival benefits of obese individuals remain unknown. Some supporting studies classify adiposity using body mass index without fully clarifying body composition parameters, which may imply that part of the protection attributed to “obesity” instead reflects preserved skeletal muscle and metabolic reserve (54, 55). Additionally, comparator groups and study designs can introduce bias. The normal-weight and underweight reference categories can be contaminated by frailty, cachexia, and chronic wasting illness, features that can intrinsically be linked to disease severity itself (56). Therefore, reverse causation can also play a role: disease-related weight loss lowers BMI, meaning a lower weight may simply indicate a more severe illness rather than cause it. Ultimately, conditioning analyses on an acute event such as ICU admission or sepsis diagnosis can further introduce index-event (collider) and survivor bias (57).

Obesity has been associated with an increased risk of recurrent, nosocomial, and secondary infections that can lead to sepsis (58), and is a significant risk factor for sepsis in COVID-19 and influenza outbreaks (59). Excess adipose tissue contributes to a chronic imbalance between high leptin levels, which have well-known pro-inflammatory properties, and low adiponectin levels, which have anti-inflammatory properties. This pro-inflammatory microenvironment and the lack of adiponectin negative feedback promote macrophage production of high levels of IL-6 and TNF-α, possibly triggering and even exacerbating the inflammatory cytokine storm seen in sepsis (59).

Furthermore, data have consistently shown the deleterious role of pre-existing sarcopenic obesity in models of immunometabolic stress, as the synergistic effects of diminished skeletal muscle and expanded visceral fat may amplify the impact on chronic inflammation, dysregulation of adipokines and myokines, and metabolic changes observed in sepsis (60). Thus, visceral obesity is closely linked to sepsis and sarcopenia pathophysiology.

### 2.5 Controversies in the sepsis-sarcopenia relationship

Despite growing evidence supporting a bidirectional relationship, several aspects of the sepsis-sarcopenia axis remain controversial. First, the “obesity paradox” in sepsis challenges the traditional view that adiposity uniformly worsens outcomes, suggesting that metabolic reserves may confer context-dependent benefits. Second, mitochondrial dysfunction in sarcopenia has been debated as either a primary driver of muscle loss or an adaptive response to reduced energetic demand. Third, the role of IL-6 remains paradoxical, acting as both a pro-inflammatory mediator in chronic disease and a regenerative myokine in exercise contexts. These contradictions underscore the need for temporally resolved and tissue-specific analyses to distinguish adaptive from maladaptive responses.

## 3 Beyond movement: immunological mechanisms underlying the sepsis-sarcopenia connection

The traditional view that the musculoskeletal system is mainly responsible for movement is shifting due to recent advances. For instance, skeletal muscle is increasingly recognized for its immunoregulatory properties. This modulating effect encompasses numerous mechanisms, including the secretion of HSPs, myokines, and other signaling molecules, as well as cell-to-cell interactions (**Figure 1B**) (61).

### 3.1 Heat shock proteins: the immuno-metabolic regulation of skeletal muscle in sarcopenia and sepsis

HSPs are molecular chaperones essential for cellular homeostasis and protecting cells against diverse stressors, including oxidative stress, inflammation, and infection (62). Beyond stress response, HSPs support key metabolic functions such as myogenic cell proliferation, insulin sensitivity (63), and cellular proteostasis (62). Because muscle-wasting conditions such as sepsis and sarcopenia are characterized by inflammation, atrophy, and weakness, understanding HSPs’ expression in skeletal muscle is essential.

Sarcopenia involves a progressive loss of muscle mass, a gradual decline in force generation, and increased susceptibility to contraction-induced injury with impaired recovery, whereas sepsis can precipitate an acute and severe form of muscle wasting (**Figure 2A**). While differing in their temporal and clinical profiles, they both converge on several shared downstream pathological mechanisms, including a diminished capacity to induce HSPs in skeletal muscle (64–66). Gene and protein expression of HSPs are reportedly downregulated with muscle disuse and inactivity (67).

Hsp72 is released into the extracellular microenvironment after tissue injury and has stress-adapting functions in skeletal muscle, as well as pro-myogenic roles in muscle development (14). It can interact with immune cell receptors in skeletal muscles to regulate the early inflammatory response. Moreover, Hsp72 is necessary for muscle fiber regrowth during regeneration and recovery following acute muscle injury, as well as for preventing sustained inflammatory lesions and persistent necrotic muscle fibers (68). Hsp72 overexpression maintains specific force, reduces markers of oxidative damage, and normalizes antioxidant enzyme levels in the muscles of old mice (69). Hsp72 may also preserve muscle mass by blocking NF-κB and Forkhead box O3 (FOXO3a) transcriptional activities and maintaining the skeletal muscle stem cells (MuSC) pool, thus downregulating the ubiquitin ligases atrogin-1 and MuRF1, which enhances structural and functional recovery and prevents muscle fiber atrophy (70).

Small HSPs, Hsp27 and αB-crystallin, are associated with actin microfilaments in developing myotubes and expressed during differentiation of myoblasts into myotubes, so Hsp27 is involved in developing skeletal muscles (71). Hsp27 protects myocytes against mechanical and oxidative stress, and overexpression of Hsp27 in rats was found to exert effects similar to those of Hsp72, thereby limiting NF-κB-driven atrogin-1/MuRF1 expression and fiber atrophy (71). Likewise, Hsp90 plays a pro-myogenic role in skeletal muscle development. The inhibition of Hsp90 leads to decreased expression of myogenic markers, such as myoblast determination protein 1 (MyoD), myogenin, AKT1, and BCL-2, which is linked to impaired myotube formation, decreased mitogenic signaling, and increased apoptosis (72).

Another key HSP is Hsp10, which helps preserve mitochondrial function, presumably by targeting abnormal proteins for degradation and preventing aberrant ROS production, thereby reducing the accumulation of oxidized proteins. Overexpression of Hsp10 in muscles of old mice prevented the decline in maximal force, preserved muscle cross-sectional area, and protected against contraction-induced damage (73).

Hence, each HSP appears to have distinct mechanisms and complex interactions among them that ultimately drive muscle growth and repair, thereby becoming relevant to understanding sepsis and sarcopenia, as well as their potential therapeutic value (65).

### 3.2 Myokines: inflammation and muscle wasting

Recent literature has recognized skeletal muscle as an endocrine organ that produces and releases soluble factors, commonly termed myokines, that exert autocrine, paracrine, and endocrine effects on numerous tissues (74). Skeletal muscle fibers produce a wide range of myokines, including IL-6, TNF-α, IL-7, IL-15, insulin-like growth factor 1 (IGF-1), IL-1β, regulated upon activation, normal T-cell expressed and secreted (RANTES), and irisin, among others (74).

IL-6 exhibits a complex biological involvement, as it can produce both pro- and anti-inflammatory effects and promote both muscle anabolism and catabolism, depending on the target structure, environment, and mode of release (11). As a pro-inflammatory cytokine, IL-6 is secreted by numerous immune cells in response to infection or tissue damage and is known to enhance T-cell recruitment and expansion, stimulate B-cell antibody production, inhibit regulatory T-cell (Treg) differentiation, and amplify lymphocyte trafficking due to upregulation of cell adhesion molecules such as ICAM-1 and CCL-2 (75). In this context, IL-6 facilitates age-related CLGI, blunts muscle anabolism and energy homeostasis, and directly promotes muscle catabolism (76).

Intriguingly, IL-6 signaling is considered crucial for skeletal muscle regeneration and hypertrophy, and circulating IL-6 concentrations increase markedly following exercise (77). It is well described that IL-6 enhances satellite cell function and glucose metabolism, promoting skeletal muscle anabolism and restoring muscle homeostasis after exercise (78). This differential response might be explained by the distinct biological environments that exercise promotes. Exercise increases intracellular Ca^2+^ levels, which in turn inhibit TNF-α expression. In parallel, calcineurin mediates IL-6 production in myocytes in response to Ca^2+^. Consequently, IL-6 is preferentially elevated in response to exercise, whereas TNF-α levels remain unchanged (79).

Thus, IL-6 synergistically interacts with co-factors such as TNF-α, amplifying its actions as a pro-inflammatory cytokine. In addition, anti-inflammatory cytokines such as IL-10 also influence the differentiation of IL-6 into an anti-inflammatory profile (79). In the context of sarcopenia and sepsis, IL-6 appears to play a pro-inflammatory role, inducing a gradual loss of skeletal muscle function (11). Nevertheless, the sole action of IL-6 alone apparently is insufficient to induce muscle mass loss in such conditions. Instead, the catabolic effect of IL-6 may depend on chronic exposure to IL-6 and on its synergistic interactions with other factors that mediate the inflammatory response, such as TNF-α (11).

TNF-α is a major driver of proteolytic pathways in sarcopenic muscle and a key pro-inflammatory mediator in sepsis, where elevated circulating levels have been associated with sepsis diagnosis and an increased risk of sepsis mortality (80). Despite its prominent and well-defined role as a pro-inflammatory cytokine, TNF-α, which functions as a myokine, is poorly recognized. Skeletal muscle fibers and myogenic cells express and secrete TNF-α, especially after injury or in myopathies (81, 82). As a myokine, it can act in an autocrine/paracrine pattern to modulate proliferation, differentiation, and fusion of muscle cells (81). The absence of TNF-α in muscle has been shown to promote myogenesis and muscle regeneration (83).

Muscle-generated TNF-α can drive local inflammation and muscle wasting, disrupting signaling between muscle and immune cells, thereby perpetuating inflammatory patterns in patients with sarcopenia and sepsis (11, 84). Muscle TNF-α may also activate NF-κB and other stress pathways in myofibers affected by aging-related sarcopenia and sepsis-associated muscle wasting, thereby driving proteolysis, impairing regeneration, and causing contractile dysfunction (85, 86). More specifically, TNF-α via NF-κB destabilizes MyoD, thereby blocking satellite cell/myoblast differentiation and inhibiting myogenesis (87, 88). It also activates the NF-κB/TRAF6-ubiquitin-proteasome axis, which, in turn, upregulates Atrogin-1 and MuRF1, promotes myosin degradation, and ultimately leads to muscle fiber atrophy (85, 86).

In sepsis, high TNF-α levels drive acute muscle wasting and weakness via the same NF-κB/ROS/ubiquitin-proteasome mechanisms and by directly causing myofilament dysfunction (89, 90). Additionally, TNF-α activates apoptosis and caspase-8/3/GSDME-mediated pyroptosis, leading to the loss of myofibers in sarcopenia (91), while an experimental sepsis model shows robust TNF-driven inflammatory and cell-death signaling in muscle (90). Thus, TNF-α acts as a maladaptive myokine that couples sarcopenia and sepsis through sustained inflammaging, impaired regeneration, catabolism, and dysfunctional cell death.

IL-7 is a recently classified myokine, due to its expression and secretion by skeletal muscle cells, that plays a vital role in the development and maintenance of immature lymphocytes and in supporting thymic function (92, 93) (**Figure 2B**). It has strong anti-apoptotic properties, inducing proliferation and re-establishing the function of CD4^+^ and CD8^+^ T cells, and improving survival in animal models of sepsis (94). In accordance, low levels of IL-7 reportedly increase mortality risk in sepsis and septic shock patients (95), while also acting as a marker for age-related muscle loss (96).

IL-15 is crucial for the development and maintenance of the immune system. It regulates the proliferation, activation, and distribution of NK-cells. At the same time, it modulates CD8 T-cell homeostasis, enhances neutrophil migration and phagocytosis, promotes B-cell proliferation, and promotes the survival of naïve T-cells (**Figure 2B**) (97). Moreover, IL-15 stimulates myogenesis, exerts a catabolic effect on adipose tissue, and promotes muscle regeneration through regulation of fibro-adipogenic progenitors (FAP) (98). A decline in serum levels of IL-15 has been observed in aging, obesity, sepsis, and sarcopenia (99, 100). So, impaired IL-15 signaling may partake in these conditions’ interdependence.

Myokines are also central mediators linking skeletal muscle and the age-dependent loss of immune function (**Figure 2B**). Increased IL-7 levels promote a thymoprotective effect in active individuals (96). While IL-7 and IL-15 levels have been shown to decrease with age in humans and mice, physical exercise effectively counteracts this decline (11, 101). Likewise, the 5′-AMP-activated protein kinase (AMPK) is pivotal for exercise-induced IL-15 production due to its role in regulating intracellular energy levels in skeletal muscle (102).

Interestingly, AMPK activity declines with age, thereby providing a potential molecular mechanism for impaired IL-15 signaling in aged muscle (103). Aging consistently leads to chronic exposure to IL-6 and the simultaneous release of pro-inflammatory cytokines, thereby amplifying the pro-inflammatory effects of IL-6 signaling and impairing IL-6 release in response to exercise, thereby reducing anti-inflammatory effects (11). These findings strengthen the connection between aging, exercise, inflammatory alterations, sarcopenia, and sepsis.

This dual role of IL-6 raises a central question: in sepsis, is its elevation an adaptive response by skeletal muscle to restore homeostasis, or does prolonged, dysregulated signaling become purely maladaptive? Rather than absolute concentration, the timing and pulsatility of myokine release now appear to be the key determinants of their pro- versus anti-inflammatory effects. Acute, transient IL-6 surges during exercise tend to be beneficial, while sustained, low-grade elevations typical of inflammaging and sepsis likely drive muscle catabolism and immune dysfunction (104). Clarifying the temporal and contextual cues that regulate the shift from adaptive to maladaptive myokine signaling remains a key research priority.

IGF-1 is a well-known anabolic growth factor that drives muscle hypertrophy by suppressing proteolysis while simultaneously accelerating satellite cell proliferation and differentiation (105). It is also known to stimulate protein synthesis by activating the AKT/mammalian target of rapamycin signaling pathway and by modulating the initiation phase of mRNA translation (105, 106). IGF-1 secretion is very sensitive to inflammatory stimuli. The function of IGF-1 is repressed under inflammatory and atrophic conditions such as sepsis and sarcopenia (107). In critically ill patients, plasma IGF-1 concentrations are decreased in both acute and chronic inflammatory states of sepsis and are inversely related to sepsis severity (108). This may be due to GH resistance, characterized by decreased cellular responsiveness to GH, leading to a decline in circulating IGF-1 and hepatic IGF-1 synthesis (109).

Furthermore, local IGF-1 administration can prevent sepsis-induced skeletal muscle wasting (110). Other mechanisms implicated in the sepsis-related decrease in circulating IGF-1 included inflammation-induced anorexia and reduced food intake, as well as increased hepatocyte nitric oxide release (111).

As for sarcopenia, repressed IGF-1 signaling plays a crucial role in inhibiting skeletal muscle protein synthesis. The activation of proteolytic pathways leads to decreased IGF-1 secretion and diminished its anabolic effect on muscle protein (112). Additional evidence suggests that plasma IGF-11 concentrations can predict muscle mass even after adjusting for confounders (113). Finally, systemic administration of IGF-1 ameliorates muscle proteolysis and increases the expression of the myogenic transcription factors involved in myogenesis and proliferation (114). These data suggest that sepsis and sarcopenia lead to decreased serum and muscle IGF-1 levels, which are associated with muscle loss.

Theoretically, administering IGF-1 would be beneficial for both conditions. However, therapeutic IGF-1 administration in septic or sarcopenic patients requires careful consideration (115). While IGF-1 enhances muscle anabolism, the use of the recombinant growth hormone in critically ill patients is not recommended due to an increased risk of infection-associated mortality (116, 117). Indeed, it has been acknowledged that pathogens may utilize host IGF-1 signaling for proliferation (118–120). These concerns highlight the need for precision nutrition models that tailor anabolic interventions, including IGF-1 supplementation, to each patient’s immunometabolic state to reduce unintended consequences and improve clinical outcomes (121).

Previous data on inflammaging suggest IL-1β as a core pro-inflammatory myokine, linked to frailty, weight loss, and muscle wasting. Skeletal muscle can generate and respond to IL-1β independent of immune cells via the NLRP3 pathway (**Figure 1B**) (19). In elderly humans and animal models, sarcopenic muscle exhibits upregulation of NLRP3, caspase-1, IL-1β, and IL-6, indicating local inflammasome activation and inflammatory degeneration (19, 122). Bovine age-related sarcopenia likewise shows increased muscle NLRP3 and elevated IL-1β (122).

Sepsis, in turn, upregulates IL-1β signaling in myocytes, thereby increasing the expression of IL-6, MuRF1, and atrogin-1, leading to myotube atrophy (123, 124). NLRP3 inhibition and knockout attenuate IL-1β production and muscle wasting, thereby protecting skeletal muscle and improving its function (123, 125). In mice and mouse models of sepsis and other inflammatory conditions, blocking upstream ROS/NLRP3 or P2X7/NLRP3 signaling reduces IL-1β, pyroptosis, and muscle loss, highlighting the role of this axis in septic and inflammatory myopathy (126, 127).

Essentially, muscle NLRP3 activation is a key common pathway between sepsis and sarcopenia, as older sarcopenic muscle with pre-activated NLRP3/IL-1β is likely more vulnerable to the IL-1β surge during sepsis, worsening ICU-acquired weakness, while sepsis episodes with robust NLRP3/IL-1β activation can accelerate long-term muscle loss, contributing to post-sepsis or late-life sarcopenia (19, 123, 124, 128, 129). This ultimately leads to shared increases in IL-1β, promoting atrophy, mitochondrial ROS, and metabolic dysfunction, creating a shared catabolic state.

CCL5, also known as RANTES, is a chemokine fundamental to the initiation and maintenance of inflammation. Skeletal muscle expresses and secretes CCL5, which in turn acts directly on muscle to induce a sarcopenic-like phenotype in myofibers (130, 131). Overexpression of CCL5 reduces muscle mass, force, fiber diameter, and levels of sarcomeric proteins in mouse muscle and myotubes (131). In cholestasis-induced sarcopenia, the diaphragm muscle shows upregulated CCL5 mRNA, linking chronic disease and muscle CCL5 to the development of sarcopenia (132). CCL5 increases oxidative stress and activates the ubiquitin-proteasome system (UPS), thereby upregulating MuRF1 and atrogin1, which have been identified as drivers of myosin degradation and muscle fiber atrophy. It also upregulates the expression of 20S proteasome subunits, as proteasome inhibition prevents CCL5-induced loss of contractile proteins and fiber atrophy. These effects are CCR5-dependent, as the CCR5 antagonist maraviroc blocks CCL5-induced sarcopenic changes (130, 131). Exercise and heat stress both reduce CCL5 expression/secretion from muscle, suggesting that lowering this myokine may be one way in which physical activity counters chronic inflammation and sarcopenia (133, 134).

In sepsis, direct data on CCL5 as a sepsis-induced myokine are lacking, but several links can be inferred. Muscle-generated CCL5 is stimulated under highly inflammatory conditions (131, 132, 135), and CCL5 from immune cells contributes to inflammatory organ injury (136). Since CCL5 promotes proteasome-dependent proteolysis in muscle and chemokines broadly impair myogenesis in sepsis, high systemic and muscle-derived CCL5 during sepsis could acutely accelerate muscle loss, particularly in already CCL5-upregulated, sarcopenic muscle. More studies are needed to support this supposition.

Lastly, irisin, a thermogenic adipomyokine derived from the transmembrane protein fibronectin type III domain-containing protein 5 (FNDC5), is known to play a protective anti-inflammatory role in inflammatory states (137). It appears to convert white to brown adipose tissue, promote autophagy, inhibit inflammatory cell migration and infiltration, mitigate endothelial barrier dysfunction and microvascular leakage, and prevent inflammasome formation, thereby reducing inflammation and enhancing cell viability (138). Irisin may serve as a diagnostic and prognostic sepsis biomarker in critically ill patients, as its levels are inversely associated with severity scores, metabolic and inflammatory markers, and adverse outcomes such as septic shock and 28-day mortality (139).

Irisin also contributes to the prevention of sarcopenia by exerting pro-myogenic effects, including promoting skeletal muscle hypertrophy, rescuing denervation-induced muscle wasting, reducing protein degradation, tissue fibrosis, and necrosis, and enhancing sarcolemmal stability (140). Additionally, aging FNDC5/irisin knockout mice exhibit accelerated loss of muscle mass and strength, whereas the delivery of recombinant irisin in aging and aged mice alleviates sarcopenia and metabolic disorders (141).

For instance, irisin may be a key mediator between sepsis and sarcopenia, modulating sepsis-related signaling pathways such as p38 mitogen-activated protein kinases and AMPK, thereby helping to suppress inflammation and protect against cellular damage (137). Interestingly, irisin also mediates adipocyte browning, neural differentiation, osteoblast proliferation, myogenic differentiation, and myoblast fusion via pathways and mechanisms that are known to be impaired in sarcopenia (142). These findings highlight the role of irisin in the mechanisms underlying sepsis and sarcopenia, in their possible prevention, and in their interaction (**Figure 1B**).

### 3.3 From cellular quality control to systemic failure: how dysregulation of proteolytic pathways drives sarcopenia-sepsis pathological crosstalk

UPS and autophagy are two major intracellular degradation pathways that maintain cellular homeostasis by removing damaged proteins, organelles, and aggregates. The UPS degrades short-lived or misfolded proteins via polyubiquitination followed by proteasomal digestion; autophagy targets long-lived proteins, aggregates, dysfunctional organelles, or pathogens for lysosomal degradation (143). These systems are tightly regulated and interconnected, forming a single proteolytic network with reciprocal compensation that ensures proteostasis under both physiological and stress conditions (143). There is extensive reciprocal regulation between the two systems: inhibition of one often upregulates the other as compensation (144).

In sarcopenic muscle, CLGI, oxidative stress, hormonal changes, and inactivity lead to persistent but mild UPS activation through Atrogin-1/MuRF-1 E3 ligases to clear damaged proteins/organelles (145). Additionally, baseline autophagy is often insufficient or dysregulated due to imbalances in the AKT-mTOR, AMPK, and FoxO pathways, leading to greater reliance on the UPS and exaggerated, prolonged muscle wasting (**Figure 1B**) (146, 147). Excessive UPS activation leads to chaperone decline and impaired proteostasis, promoting the accumulation of misfolded/aggregated proteins, damaged organelles, oxidative stress, and impaired regeneration through suppression of MyoD and myogenin transcriptional activity and depletion of the MuSC pool (**Figure 1B**), thereby contributing to progressive fiber atrophy and weakness (148).

Sepsis concurrently activates both autophagy and the UPS, and their coordinated action initially ensures efficient clearance of damaged proteins and organelles, a process critical for skeletal muscle integrity during catabolic states (149). UPS is often the final common pathway for much of sepsis-induced proteolysis, such as NF-κB/FoxO-induced upregulation of the E3 ligases Atrogin-1/MAFbx and MuRF1, which ubiquitinate myofibrillar and metabolic proteins for proteasomal degradation (150, 151). Autophagy is rapidly induced in skeletal muscle during sepsis, driven largely by AKT-mTOR inhibition and AMPK activation, with increased LC3-II, autophagosome formation, and autophagy gene expression (149, 150). Functionally, muscle autophagy is protective rather than purely catabolic. Autophagy helps clear dysfunctional mitochondria and protein aggregates. Its blockade in sepsis leads to mitochondrial damage and long-term weakness, which can be improved by restoring autophagy flux (152).

Nevertheless, dysregulation of these pathways during sepsis not only exacerbates muscle wasting but also influences communication between skeletal muscle and other organs through metabolic, inflammatory, and signaling mediators (9). For example, loss of autophagy in sepsis drives stronger UPS activation and more severe atrophy, indicating compensatory crosstalk between the two systems (147). Chronic overactivation of these systems within skeletal muscle, common in prolonged sepsis, may also drive pathological muscle wasting through alterations in immune cell function and defective autophagic flux, which can exacerbate multi-organ failure seen in critically ill sepsis patients (153, 154). This process, termed “septic autocannibalism”, while detrimental to long-term muscle integrity, is understood as a metabolic defense priority that ensures the availability of amino acids for critical immune and systemic functions during acute stress (10, 47).

UPS and autophagy are also key pathways in the interplay between sarcopenia and sepsis, as pre-existing sarcopenia means baseline chronic, lower-grade UPS overactivity, and sepsis superimposes a sharper, more acute catabolic surge, driving exaggerated Atrogin-1/MuRF1 and proteasome activation and more severe muscle loss (151). Also, chronic UPS overactivation suppresses basal autophagy in skeletal muscle in sarcopenia, thereby disrupting its protective role in sepsis and leading to loss of muscle mass, quality, and function (147, 149).

## 4 Metabolic signatures of sepsis and sarcopenia: mitochondrial disruption as a central metabolic signature in systemic inflammation and muscle loss

Sepsis and sarcopenia share a convergent metabolic foundation where mitochondrial dysfunction acts as a central nexus, explaining how each condition exacerbates the other. This dysfunction triggers proteolytic pathways in skeletal muscle, driving atrophy while simultaneously impairing immune responses. Furthermore, the interplay between mitochondrial failure and systemic inflammation leads to disorganized mobilization of energy substrates, specifically lipids and amino acids, with profound clinical consequences. This metabolic interplay could be further complicated by the metabolic defense priority described above, whereby lipids are preferentially mobilized to spare muscle protein. However, in the context of pre-existing sarcopenia or adipose tissue dysfunction, this protective mechanism can fail, leading to accelerated muscle wasting (47).

During the acute phase of sepsis, human energy metabolism undergoes radical metabolic reprogramming, characterized by upregulation of glycolysis and concurrent downregulation of oxidative phosphorylation (OXPHOS) (155). While this shift addresses the immediate energy requirements of activated immune cells, it is energetically inefficient, resulting in a significantly reduced intracellular ATP/ADP ratio (156). As the inflammatory response progresses, this “adaptive” shift often transitions into mitochondrial failure (157). For the sarcopenic patient, this acute crisis impacts an already compromised system. Sarcopenic muscle exhibits pre-existing age-related mitochondrial decay, characterized by the downregulation of genes involved in OXPHOS (e.g., *ERRα* and *PGC-1α*) and NAD^+^ biosynthesis (158, 159). Beyond genetic signatures, functional evidence from magnetic resonance spectroscopy reveals mitochondrial inefficiency in these patients, as evidenced by a higher ATP cost per contraction despite matched contractile power (strength/cross-sectional area) (160). Furthermore, lower oxidative enzyme activity (i.e., citrate synthase, SDH, and complex III) has been associated with a lower fractional contribution of OXPHOS, identifying these enzymes as potential contributors to mitochondrial inefficiency in sarcopenic muscle (160).

While this inefficiency is often viewed as a primary defect, it may also represent an adaptive response to decreased energy demand in atrophic muscle - a “defect versus adaptation” theory that remains a critical area for future study. This is particularly relevant, given that exercise, which increases energy demand, is the most validated intervention for these pathways, although the precise mechanisms by which exercise exerts its effects are not fully understood (161). In this regard, exercise is key to mitigating the adverse metabolic consequences of obesity, but as a standalone intervention, it may not be sufficient to treat the disease (162). Therefore, future studies aimed at decoding how exercise influences mitochondrial health will provide new insights into the “defect versus adaptation” theory of mitochondrial dysfunction in sarcopenia and sepsis. Other therapies, such as gene therapy or mitochondrial transplantation, are also under investigation but require further validation (159, 163).

### 4.1 Mitochondrial quality control disruption in sepsis and sarcopenia

In these models, central to this nexus is the disruption of mitochondrial quality control (MQC), a coordinated system of mitochondrial dynamics (fusion and fission), mitophagy, and biogenesis (159, 164). MQC is indispensable for physiological homeostasis, ensuring efficient energy production and substrate mobilization while providing a vital buffering capacity. By sequestering calcium and preventing the cytosolic leakage of ROS, mitochondrial DNA (mtDNA), and other damage-associated molecular patterns (DAMPs), functional MQC preserves systemic metabolic integrity. However, in both sepsis and sarcopenia, these regulatory processes collapse, precipitating a breakdown in mitochondrial and systemic function (154, 163).

#### 4.1.1 Impairment of fusion and fission in sarcopenia and sepsis: from gradual decay to acute crisis

The regulation of mitochondrial function relies fundamentally on a continuous cycle of mitochondrial network remodeling, known as mitochondrial dynamics (165). This process is governed by a balance between fusion, mediated by MFN1/2 and OPA1, which repairs damaged organelles by exchanging genetic and metabolic components, and fission, regulated by DRP1 and FIS1, which isolates damaged segments for degradation (166, 167). When this equilibrium is disrupted, mitochondria become fragmented or dysfunctional, thereby reducing oxidative capacity and leading to subsequent muscle atrophy (168).

In the context of aging and sarcopenia, mitochondrial dynamics shift toward a dysfunctional equilibrium. Evidence suggests that MFN2 levels are a primary determinant of muscle mass; while MFN2 deletion in aged muscle can induce a sarcopenic phenotype, its overexpression has been shown to trigger mild hypertrophy (165, 169). Despite these experimental findings, clinical data remain heterogeneous, with some cohorts showing reduced MFN2/OPA1 expression in older adults and others reporting no clear differences in protein expression (170). Fission is equally critical but sensitive to precise regulation in aged tissue. Experimental models demonstrate a “Goldilocks” effect for DRP1, in which both overexpression and knockdown lead to atrophy and mitochondrial dysfunction (171). Previous data indicate decreases in DRP1, Fis1, and other fission proteins in aging/sarcopenia, leading to impaired mitophagy and accumulation of dysfunctional mitochondria (165, 172). However, animal data suggest that reduced fission may occur even without major changes in total DRP1 (173). Lifestyle factors such as caloric restriction and exercise tend to partially normalize age−related fission/fusion protein changes (170, 174).

Unlike the gradual decline seen in sarcopenia, sepsis (e.g., CLP, endotoxemia) triggers an acute and pronounced shift toward a fission-dominant state. This rapid imbalance results in severe mitochondrial fragmentation, characterized by downregulation of fusion proteins (MFN2, OPA1) and upregulation of fission markers (DRP1, FIS1) in skeletal muscle and multiple organs (152, 175). This disruption is organ-dependent, being particularly severe in limb muscles compared with the diaphragm, and directly correlates with ICU-acquired weakness (176). A key mechanistic bridge between septic inflammation and muscle loss is the S100A8/A9-RAGE–Drp1 signaling pathway (177). In CLP models, this pathway drives Drp1 phosphorylation, mitochondrial fragmentation, ATP loss, and muscle atrophy, factors that were mitigated with pharmacological inhibition with Mdivi-1 (10 mg/kg for 3 days post−CLP) (177, 178). Nonetheless, it is important to highlight that these findings are primarily pre-clinical and that complete DRP1 knockdown can itself induce severe atrophy, suggesting that therapeutic strategies in sepsis-sarcopenia conditions should likely focus on partial and time-limited inhibition (179).

#### 4.1.2 Mitophagy and biogenesis in sarcopenia and sepsis

Under physiological conditions, mitochondrial health is maintained by a balanced turnover cycle: mitophagy selectively removes damaged fragments isolated by fission, while biogenesis replenishes the pool to preserve metabolic capacity. In both sepsis and sarcopenia, this homeostatic cycle collapses, leading to an accumulation of aberrant mitochondria that exacerbate oxidative stress and muscle wasting.

In healthy cells, mitophagy is primarily regulated through ubiquitin-dependent or ubiquitin-independent pathways. The canonical ubiquitin-dependent route is the PINK1/Parkin pathway (180). PINK1 is an outer membrane protein that, upon activation, recruits the cytosolic ligase, Parkin. Parkin then ubiquitinates the mitochondrion, signaling the cellular autophagy machinery (180, 181). Alternatively, mitophagy can occur independently of ubiquitin or Parkin through receptor-mediated mechanisms involving BNIP3/NIX, FUNDC1, and Bcl2-L-13 (182). Complementing this clearance, mitochondrial biogenesis is orchestrated by transcriptional coactivators such as PGC-1α, which activates NRF1/2 to increase TFAM expression (183). Ultimately, TFAM drives the replication and transcription of mitochondrial DNA (mtDNA), thereby enabling the formation of new functional mitochondria (184). These two processes are intrinsically linked: mitophagy often triggers compensatory biogenesis, while impaired mitophagy may increase the accumulation of dysfunctional organelles through impaired clearance (185).

These pathways represent a critical nexus between metabolic state, immune stress, and lifestyle factors. For instance, a high AMP/ATP ratio activates AMPK, an energy sensor that simultaneously stimulates biogenesis via PGC-1α and promotes mitophagy via ULK1, which phosphorylates Parkin, FUNDC1, and BNIP3 (186–188). Additionally, the AMPK/p38/PGC-1 axis can be activated by oxidative stress (via NRF2), calcium signaling, and environmental stimuli such as exercise and cold exposure (189–191).

In sepsis, mitophagy is impaired through several convergent mechanisms. Primarily, NLRP3 inflammasome activation upregulates caspases that cleave Parkin and degrade PINK1, leading to the accumulation of damaged mitochondria (164). This dysfunction is further exacerbated by TLR4 signaling, which suppresses AMPK activation (192, 193). In immune cells, particularly macrophages, prolonged NLRP3 activation remains a key driver of mitophagic failure (a process notably regulated by the stress-inducible protein SESN2) (194). Beyond these signaling cascades, sepsis disrupts endosomal-dependent clearance by depressing SIRT1 activity (a protein that acts as an NAD^+^-dependent deacetylase) (195). This occurs because systemic inflammation triggers a massive depletion of NAD^+^, the essential substrate for SIRT1 function (196).

Mitophagy via receptor-mediated mechanisms also appears to be impaired, with prior data indicating that, at the transcript level, FUNDC1, BCL2L13, FKBP8, and PHB2 are downregulated, suggesting a net failure of the receptor pathway (154). Biogenesis is also impaired in sepsis, with elevated pro-inflammatory cytokines in early-phase sepsis and sarcopenia (e.g., TNFα) downregulating PGC-1α expression, thereby reducing biogenesis and impairing TFAM translocation (197). As sepsis progresses, there is also evidence of compensatory biogenesis mechanisms that are often insufficient or maladaptive, leading to the accumulation of aberrant mitochondria that exacerbate ROS production and organ dysfunction (197).

Similar deficits are described in sarcopenic patients. In COPD-related sarcopenia, reduced Parkin-mediated mitophagy drives muscle atrophy via ROS–MuRF1 signaling (198). In aging skeletal muscle, while BNIP3 typically increases as a protective response to maintain flux, deficiencies in this axis are linked to NLRP3-mediated inflammation and atrophy (199). Notably, the BNIP3 axis appears to be a major mitophagic pathway that becomes impaired in sarcopenic patients (200). Biogenesis is also profoundly affected: cohorts of older adults with sarcopenia exhibit a distinct transcriptional signature of bioenergetic failure, characterized by downregulated PGC-1α/ERRα signaling, OXPHOS, and proteostasis genes (158). Notably, sedentary individuals exhibit ~50% lower PGC-1α and SIRT3 levels than younger controls, and these levels directly correlate with diminished physical performance (201). Similar to impairments in fusion and fission processes, these age-related mitochondrial deficits can be ameliorated by exercise, which serves as a key mechanism for restoring homeostasis by activating the AMPK/SIRT1-PGC-1α pathway (202). Consequently, pharmacological agents that boost PGC-1α-dependent biogenesis hold significant therapeutic potential to enhance muscle metabolism and proteostasis in aging models (203, 204).

#### 4.1.3 Mitochondrial calcium buffering dysfunction as a driver of inflammatory proteolytic signaling

The disruption of calcium signaling represents another key nexus linking mitochondrial dysfunction to tissue damage in both sepsis and sarcopenia. Under physiological conditions in resting muscle, mitochondria function as dynamic calcium buffers through mitochondrial calcium uniporters, thereby preventing cytosolic calcium overload (205). However, in hypermetabolic stress models such as sepsis and sarcopenia, mitochondrial dysfunction, coupled with a low ATP/AMP ratio, limits energy-dependent calcium uptake, thereby playing a central role in disrupting intracellular calcium homeostasis (206). Consequently, excess cytosolic calcium can activate NLRP3 inflammasome and cytosolic enzymes, including the protease calpain, as well as phospholipases and endonucleases (207). This response can trigger widespread cellular and nuclear damage, including myocyte injury (exacerbating sarcopenia) and damaging other tissues (a hallmark of sepsis). Under these conditions, detrimental signaling from sustained elevations in intracellular calcium levels is also driven by other factors beyond impaired energy metabolism (208). Increased ROS and inflammatory cytokines also affect various calcium channels, including voltage-gated, receptor-operated, and store-operated channels, and impact the expression of calcium pumps in the endoplasmic reticulum (208).

Other inflammatory pathways linked to proteolysis are closely associated with mitochondrial dysfunction. Mitochondrial dysfunction is not merely a consequence of systemic inflammation. It is a primary driver of the inflammatory proteolytic pathways that accelerate myofibrillar protein degradation. These inflammatory cascades, for instance, the NLRP3 inflammasome and NF-κB are tightly coupled to mitochondrial integrity (164, 209). Furthermore, the cytosolic release of mtDNA and ROS acts as a potent endogenous stimulus for NLRP3 inflammasome activation (210). Simultaneously, this pro-inflammatory milieu (TNFα, IL-6, and IL-1β) feeds the NF-κB-Atrogin-1/MuRF1 proteolytic axis described above (25). Ultimately, this transforms mitochondria from stress-resilient energy producers into inflammatory stressors and proteolytic triggers. The result is a self-perpetuating vicious cycle: damaged mitochondria release DAMPs and promote cytokine-mediated activation of proteolytic systems, leading to the muscle atrophy observed in both acute septic patients and the chronic sarcopenic elderly.

### 4.2 Disorganized energy mobilization: the amino acid and lipid shifts

The resulting mitochondrial crisis, marked by reduced OXPHOS and a high AMP/ATP ratio, activates AMPK, which induces a catabolic survival program. In this context, AMPK functions as a key cellular energy sensor, promoting catabolic pathways and inhibiting anabolic processes to sustain ATP production. AMPK influences and is influenced by different myokines and is known to suppress the Warburg effect (aerobic glycolysis) in monocytes and macrophages, thereby counteracting the elevated glycolytic activity and reducing the release of pro-inflammatory mediators such as HMGB1 in mice models of sepsis (211, 212).

Numerous studies have demonstrated a protective role for pharmacological activation of AMPK during sepsis, highlighting its anti-inflammatory properties, antimicrobial effects, and its capacity to enhance mitochondrial biogenesis and autophagy (213). However, while AMPK exerts a protective immunometabolic regulation and emerging evidence suggests it may even protect myocytes, the overall dysregulation and overactivation of catabolic pathways in these patients can deplete their energy reserves. This dysregulated response in sepsis can mimic what has been called a failing “starvation state response” (214).

**Figure 3:**
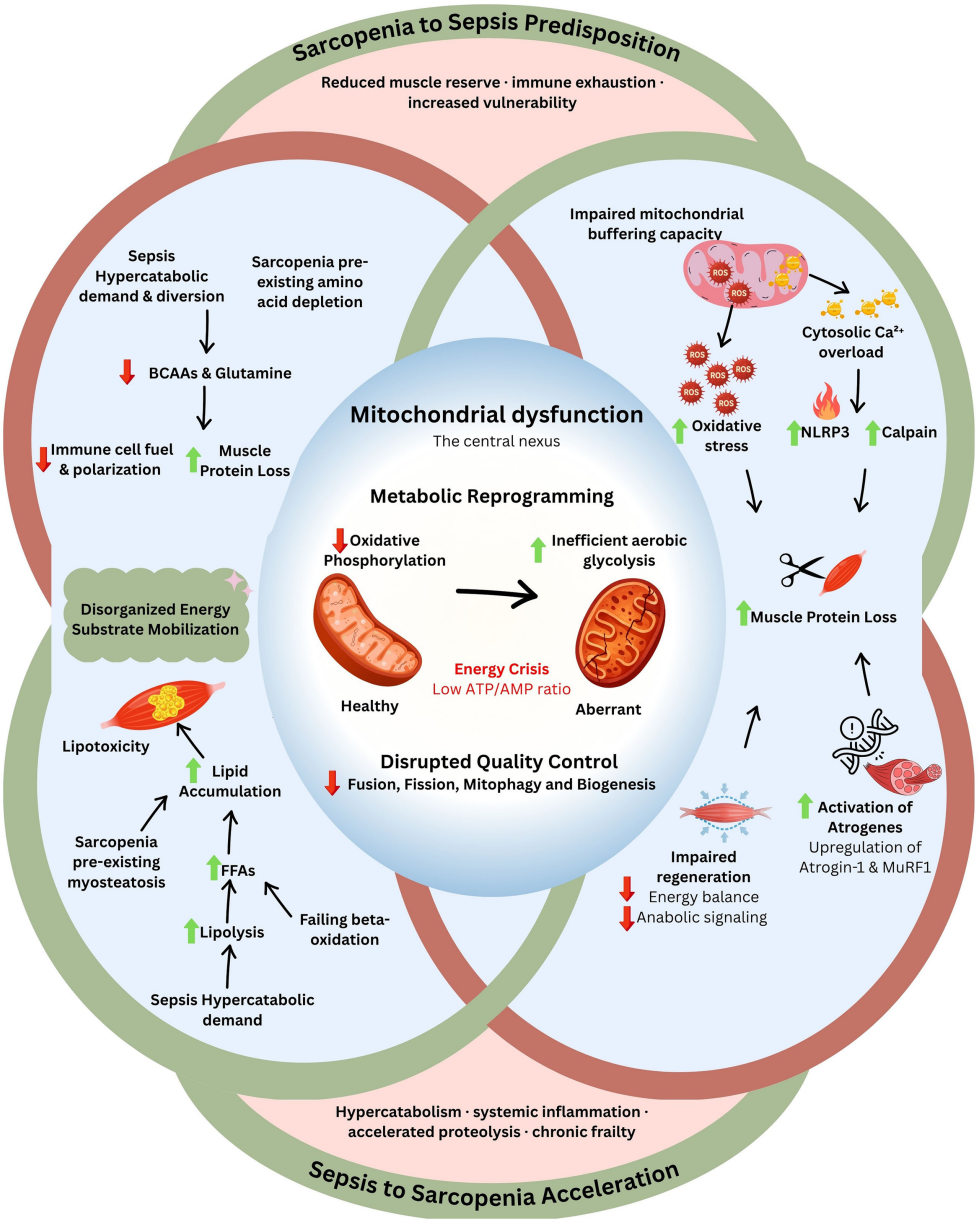
The bidirectional interplay between sarcopenia and sepsis: mitochondrial dysfunction as the central mechanistic nexus. Central to this pathophysiology is mitochondrial dysfunction, which drives metabolic, proteolytic, and inflammatory collapse. At the core lies mitochondrial dysfunction, with metabolic reprogramming from oxidative phosphorylation toward inefficient aerobic glycolysis, an energy crisis (low ATP/AMP ratio), and disrupted quality control (impaired fusion, fission, mitophagy, and biogenesis), shared by both conditions and contributing to both effector arms. *Right lobe - proteolytic and inflammatory cascade.* Impaired mitochondrial calcium buffering produces cytosolic Ca^2+^ overload, activating calpain and the NLRP3 inflammasome and increasing oxidative stress. This, together with activation of the atrogenes atrogin-1 and MuRF1 and impaired regeneration (reduced energy balance and anabolic signaling), converges on muscle protein loss. *Left lobe - disorganized energy-substrate mobilization.* In the amino-acid arm, sepsis-driven hypercatabolic demand and diversion, together with pre-existing sarcopenic amino-acid depletion, lower circulating BCAAs and glutamine, impairing immune-cell fuel and polarization and contributing to muscle protein loss. In the lipid arm, sepsis-driven lipolysis and failing β-oxidation raise free fatty acids (FFAs), which, combined with pre-existing sarcopenia and myosteatosis, promote intramuscular lipid accumulation and lipotoxicity. The central nexus and the two lobes depict shared mechanisms, and the relative strength of evidence for individual pathways is summarized in **Table 1**. AMP, adenosine monophosphate; ATP, adenosine triphosphate; BCAAs, branched-chain amino acids; FFAs, free fatty acids; MuRF1, muscle RING-finger protein-1; NLRP3, NLR family pyrin domain containing 3.

Consequently, in sepsis, there is a disorganized mobilization of energy source molecules (**Figure 3**). This includes protein breakdown and the mobilization of triacylglycerides, which yield gluconeogenic substrates such as amino acids, glycerol, and FFAs (215). Indeed, sepsis-induced muscle loss is characterized by excessive degradation of structural proteins, particularly myosin heavy chains, which predominantly leads to the atrophy of type II fibers (9). Although the heightened protein breakdown can generate free amino acids, the progressive decline in muscle mass may ultimately reduce aminoacidemia (216).

**Table 1:** Major pathways linking sarcopenia and sepsis.

Pathway/mechanism	Predominant level of evidence	Clinical relevance	Key reference(s)
Heat shock proteins (Hsp72, Hsp27, Hsp90, Hsp10)	Mainly preclinical (rodent) and *in vitro* (myotube); limited human data (downregulation with disuse/inactivity)	Candidate targets to preserve muscle mass and limit atrophy	(14, 65, 68–73)
IL-6 (dual pro-/anti-inflammatory myokine)	Human (exercise and sepsis cohorts) and preclinical	Context-dependent: acute exercise surgest beneficial, chronic elevation catabolic; candidate biomarker/target	(74–78)
TNF-α (myokine; NF-κB–atrogene axis)	Elevated circulating TNF-α in human sepsis (observational); Anti TNF-α therapy potentially beneficial in sepsis (meta-analysis) muscle-derived myokine actions mainly preclinical	Associated with sepsis severity/mortality; driver of proteolysis	(80, 81, 83, 85–88, 91, 266, 267)
IL-7 and IL-15 (immunoregulatory myokines)	Human observational (low IL-7 ↔ sepsis mortality; serum IL-15 declines with age/obesity/sepsis/sarcopenia) plus preclinical	Candidate prognostic markers and immunomodulatory targets	(94, 95, 97, 99, 100)
IGF-1 (anabolic myokine)	Human (reduced plasma IGF-1 in critical illness, inverse with severity) plus preclinical (local IGF-1 prevents wasting)	Marker of anabolic resistance; potential target	(108, 110, 111, 113)
IL-1β/NLRP3 inflammasome	Human (NLRP3/IL-1β upregulation in aged/sarcopenic muscle) plus preclinical (animal sarcopenia/sepsis models; NLRP3 inhibition/knockout)	Convergent node linking sarcopenia and sepsis; potential target	(19, 122–125, 128, 164)
CCL5 (RANTES)	Sarcopenia: preclinical/*in vitro* (mouse muscle, myotubes; CCR5-dependent). Sepsis: inferred — direct data lacking	Hypothesized contributor to muscle loss; speculative in sepsis	(130–134, 136)
Irisin (FNDC5-derived adipomyokine)	Human (inverse association with sepsis severity/mortality) plus preclinical (pro-myogenic; FNDC5 knockout/recombinant irisin)	Candidate diagnostic/prognostic biomarker; protective myokine	(138–142, 268)
Ubiquitin–proteasome system and autophagy (Atrogin-1/MuRF1, FoxO, LC3)	Preclinical studies, human observational studies	Final common pathway of muscle proteolysis	(143–147, 149–153)
Mitochondrial dynamics (fusion/fission; MFN2, OPA1, DRP1, FIS1)	Preclinical (CLP/endotoxemia; knockout/overexpression; Mdivi1); human cohort data heterogeneous	Links septic inflammation to ICU-acquired weakness	(165, 167, 169–171, 175, 176, 178, 179)
Mitophagy and biogenesis (PINK1/Parkin, BNIP3, PGC-1α, SIRT1, TFAM)	Mostly preclinical studies, few evidence regarding observational studies	Influence on mitochondrial quality and energetic capacity	(154, 158, 164, 183, 185, 191, 193, 194, 197, 201)
Mitochondrial Ca^2+^ dysregulation and inflammatory proteolysis (calpain; mtDNA/ROS–NLRP3/NF-κB)	Mostly preclinical studies	Couples mitochondrial failure to proteolysis and DAMP release	(205–207, 209, 210)
Amino-acid reprogramming (BCAAs, glutamine, alanine)	Human (plasma amino-acid changes; glutamine as post-operative sepsis predictor; large community BCAA cohort) plus preclinical	Substrates for immune function/gluconeogenesis; nutritional targets	(217, 219, 220, 224–226, 232, 265)
Metabolite-mediated injury (kynurenine/IDO-1, lipotoxicity/FFA, hyperammonemia–myostatin)	Mixed — human (IDO-1 ↔ sarcopenia/mortality in sepsis; FFA/albumin; ammonia in muscle-depleted cirrhosis) plus preclinical/*in vitro* (kynurenine myoblast models)	Potential biomarkers and contributors to muscle loss/central fatigue	(236, 240, 241, 244, 249, 255, 257, 259, 262)
Physical activity/exercise	Strong human (epidemiological and interventional) plus preclinical	Most validated preventive/therapeutic intervention	(26, 28, 30, 31)

Overall, septic and sarcopenic patients exhibit impaired amino acid metabolism, with progressively diminished stores of crucial amino acids, including branched-chain amino acids (BCAAs; leucine, isoleucine, and valine) and glutamine (217). For instance, a recent community-based study involving more than 100, 000 older participants (mean age: 56.40 ± 8.09 years) demonstrated that BCAAs were positively correlated with muscle mass and handgrip strength in sarcopenia, and that higher valine concentrations were associated with a decreased risk of developing sarcopenia (218).

BCAAs are crucial amino acids for muscle mass maintenance. However, studies on their role in sarcopenia and sepsis often appear contradictory, suggesting that BCAAs have mixed effects on these conditions (219). These divergent results may, in part, reflect differences in the underlying metabolic context and in the compartmentalization of BCAA depletion or accumulation across these diseases. BCAAs are known to activate mTOR signaling in skeletal muscle fibers, thereby promoting protein synthesis and reducing protein breakdown. (217). However, in the context of BCAA overaccumulation in skeletal muscle, followed by sustained mTOR activation, this ultimately impairs insulin sensitivity, inhibits autophagy, and disrupts mitochondrial function, leading to excessive muscle wasting (220). In this context, sarcopenic patients show intramuscular accumulation of BCAAs and reduced plasma concentrations, which impair their ability to maintain muscle mass. Because both excessive and insufficient mTOR activation can cause muscle wasting, tightly regulated BCAA metabolism is crucial for preserving muscle mass.

Nonetheless, it is essential to recognize that the role of BCAAs in preserving muscle mass extends beyond the mTOR pathway, encompassing significant immunomodulatory effects as well (221). For instance, BCAAs can activate the PI3K/Akt pathway, promoting cell survival and reducing inflammation-induced muscle damage (222). Indeed, BCAAs have been widely investigated in relation to the immune response. BCAAs play a role in immune cell metabolism, acting as fuel for rapidly proliferating cells and supporting their biosynthetic needs (221). Through the mTORC1 and JAK/STAT pathways, BCAAs promote immune cell growth, differentiation, and cytokine production and signal transduction, all of which are essential for effective immune defense (221). Additionally, BCAAs appear to play an immunoregulatory role, preventing the excessive release of IL-1β and IL-18 by modulating NLRP3 inflammasome activity (223). Thus, it is reasonable to suggest that depleted BCAA stores in sarcopenic patients can impair immune function (**Figure 2B**). In contrast, the sepsis-induced consumption of BCAAs may impact normal mTOR activation, leading to muscle wasting.

A similar rationale can also be applied to glutamine, the most abundant plasma amino acid, which has been shown to regulate protein synthesis and skeletal muscle mass (224). Significantly, the BCAA role as anabolic inducers in myocytes via mTOR is impaired when glutamine is unavailable, suggesting a crucial role for glutamine in sepsis and sarcopenia (225). Plasma glutamine concentrations have been reported to be lower in pre-operative sarcopenic patients, being an independent risk factor for predicting sepsis in post-operative patients (226). Addressing impaired glutamine metabolism is vital because glutamine is a key immunonutrient that influences energy metabolism, inflammation, and oxidative stress. Beyond glutamine itself, its metabolites can also shape immunometabolism. For example, the deamination of glutamine to α-ketoglutarate induces M2 macrophage polarization, whereas a low α-ketoglutarate/succinate ratio promotes pro-inflammatory activity (227). Similarly, arginine and metabolites associated with its metabolism can affect M1/M2 polarization in macrophages (228, 229).

To further elucidate the complexity of amino acid signaling mechanisms in septic-sarcopenic patients, it is essential to consider the concomitant exacerbation of lipid mobilization, as previously mentioned. In these patients, the hypercatabolic state stimulates the mobilization of fatty acids from adipocytes within a metabolic environment characterized by insulin resistance. Importantly, this lipid mobilization represents a key interface between metabolic and immune responses, as inflammatory stimuli also induce lipolysis via activation of the IRE1 kinase (230). This process, along with impaired lipid oxidation mechanisms during sepsis (such as PPAR-alpha dysfunction), increases circulating FFA, which may exceed energy requirements or mitochondrial metabolic capacity (231, 232). Excess of fatty acids can be re-esterified into triglycerides, leading to their accumulation in tissues such as the liver and muscles, which can result in tissue damage and potentially worsen preexisting sarcopenia (233).

In addition, excess FFAs compete for binding sites on albumin, a negatively regulated acute-phase protein that declines in models of systemic inflammation and metabolic stress. Critically ill patients also present high levels of FFAs and lower albumin levels, which saturate albumin and correlate with poor outcomes (234, 235). This decreased albumin binding capacity can displace albumin-bound amino acids, thereby increasing their free forms in circulation (236).

Aromatic amino acids (tryptophan, phenylalanine, and tyrosine) are essential for neurotransmitter synthesis and are influenced by both peripheral and central metabolism (237, 238). Among them, tryptophan is a key amino acid that can be released into free form due to reduced binding capacity of blood albumin, and its metabolism has been linked to both inflammation and muscle loss. Free blood tryptophan is primarily metabolized via the tryptophan-kynurenine pathway (KP), which has been increasingly linked to the inflammatory response. Indoleamine 2, 3-dioxygenase 1 (IDO-1), an enzyme that participates in tryptophan conversion to kynurenine, can be activated by IL-6 and potentially facilitates muscle fiber degradation (239). In mouse and human myoblast models, it has been hypothesized that kynurenine can stimulate oxidative stress and lipid peroxidation by activating very long-chain acyl-CoA dehydrogenase, thus reducing muscle fiber size and strength (240). An increase in IDO‐1 activity is associated with increased sarcopenia and mortality in septic patients (241).

Moreover, not only can KP be stimulated by inflammation and contribute to the development or worsening of sarcopenia, but sarcopenia itself can also influence the KP. Sarcopenic patients exhibit elevated levels of kynurenine and an increased kynurenine/tryptophan ratio (242). Frailty and physical disability in sarcopenic individuals lead to decreased exercise, which worsens their condition. Physical activity is known to modulate the KP in beneficial ways, making regular movement crucial for managing sarcopenia (243). Thus, KP metabolites have garnered increasing scientific attention for their role in mediating inflammation and muscle loss in older adults. Recently, an *in vitro* study using cell models demonstrated that IDO1 inhibitors may have therapeutic potential for managing septic cytokine storm (244).

While systemic inflammation can elevate the unbound fraction of tryptophan, its overall availability depends on a complex balance of tryptophan metabolic pathways (such as the kynurenine and serotonin routes, dietary intake, gut microbiome metabolism, and central uptake (236). Consequently, an increase in this free fraction may accelerate both tryptophan metabolism and central uptake, potentially leading to systemic tryptophan depletion (245). This increased central uptake is driven by the competition between free tryptophan and BCAAs for transport across the blood-brain barrier (BBB), and provides substrate for enhanced central serotonin (5-HT) synthesis, a proposed key contributor to central fatigue (246–248). The same central fatigue rationale has been applied to the competition between all aromatic amino acids and BCAA, an interaction defined as the Fischer ratio, which was initially investigated in liver failure but has since been expanded to other models of metabolic stress (249–251). In clinical practice, central fatigue is a hallmark of various inflammatory diseases and is frequently associated with low mood, lethargy, and depression (252, 253). In patients with sepsis and sarcopenia, these symptoms not only increase mortality risk but also impair the motivation required to adhere to the lifestyle interventions essential for preventing both conditions (254).

Blood ammonia represents another critical link between sepsis and sarcopenia, and is also associated with neurotoxicity, central fatigue, and encephalopathy (255). Sepsis is known to induce liver dysfunction, impairing the organ’s metabolic capacity and reducing the conversion of ammonia to urea, potentially leading to toxic hyperammonemia (215, 256). Sarcopenia can exacerbate this condition because skeletal muscle serves as a vital secondary site for ammonia buffering via the expression of glutamine synthetase (EC 6.3.1.2), an enzyme also highly expressed in hepatocytes (257). This secondary clearance mechanism explains why higher blood ammonia concentrations are observed in cirrhotic patients with significant muscle depletion (258). In a reciprocal interplay, sepsis-induced hyperammonemia can also exacerbate muscle wasting through various mechanisms. For instance, elevated ammonia concentrations can activate myostatin, thereby inhibiting protein synthesis and muscle anabolism (259). This disruption, in turn, can lead to the intramuscular accumulation of BCAAs, leading to abnormal mTOR signaling and insulin resistance (260).

Regarding lipid metabolism, our group has recently explored how lipid oxidation dysfunction plays a significant role in sepsis progression, which is why it was not discussed in depth in this review (215). Instead, we highlight that changes in lipid mobilization in patients with sepsis can occur concurrently, affect amino acid metabolism, and contribute to lipotoxicity and myosteatosis. Additionally, lipids have been implicated in fundamental immunometabolic changes, including insulin resistance and neurotoxicity (261). For example, these conditions can increase ceramide synthesis, leading to insulin resistance, while lipotoxicity may impair hepatic and muscle glucose regulation and lead to muscle damage (262). In a reverse way, sarcopenia can also drive insulin resistance, leading to an impaired immunological response.

Overall, it is evident that impairment of energy metabolism underlies many of the metabolic consequences observed in both sepsis and sarcopenic patients (**Figure 3**). The sepsis hypermetabolic state, combined with impaired mitochondrial function, features a metabolic signature that is even more relevant for sarcopenic patients, as it contributes to decreased skeletal muscle mass, increased overall fatty deposition in skeletal muscle, frailty, and depressed energy maintenance (9). Thus, exercise is crucial for enhancing metabolic health by promoting mitochondrial biogenesis and muscle growth, inducing myokine production, thereby potentially contributing to the mitigation of age-related muscle dysfunction, including sarcopenia (263) and sepsis.

Beyond mitochondrial dysfunction, extensive reprogramming of amino acid flux is a critical yet often overlooked contributor to immunometabolic decline in sepsis and sarcopenia. In sepsis, skeletal muscle rapidly undergoes proteolysis, releasing large amounts of amino acids, especially glutamine and alanine, into the circulation. This process is a coordinated but ultimately maladaptive attempt to provide essential substrates. Glutamine, in particular, supports lymphocyte proliferation, macrophage function, and glutathione synthesis, all of which are vital for antioxidant defense (15, 264). Alanine is a key substrate for hepatic gluconeogenesis, helping maintain glucose homeostasis during the hypermetabolic stress response (265). However, sustained amino acid efflux from skeletal muscle drives severe muscle wasting, leading to long-term functional impairment and increased mortality. Clarifying the regulatory mechanisms that govern this redistribution and developing strategies that preserve muscle protein while maintaining immune function represent both a major challenge and a promising therapeutic opportunity.

Whether mitochondrial dysfunction constitutes a primary pathogenic driver or a secondary adaptive response remains unresolved. Growing evidence supports a “maladaptive adaptation” model, whereby early metabolic reprogramming initially serves protective immune functions but becomes harmful when prolonged. Consequently, timing emerges as a critical determinant of therapeutic efficacy: interventions targeting restoration of oxidative metabolism are likely to be effective only within defined temporal windows of sepsis progression.

## 5 Future research avenues

Several critical mechanistic gaps continue to constrain a causal understanding of the sepsis–sarcopenia axis and impede therapeutic development. A central unresolved question is whether skeletal muscle–derived signals are necessary drivers of systemic immune dysregulation in sepsis or are simply permissive. While altered myokine profiles are associated with immune dysfunction, it remains uncertain whether skeletal muscle actively shapes immune responses or predominantly mirrors systemic inflammation.

Clarifying this will require inducible, muscle-specific loss- and gain-of-function models targeting mediators such as IL-6, IL-15, or PGC-1α in clinically relevant sepsis models, combined with single-cell profiling of immune compartments. These studies could determine whether muscle-derived signals reprogram immune cell phenotypes or primarily adjust response magnitude.

Whether pre-existing sarcopenia primes inflammasome activation and reshapes inflammatory set points remains unresolved. Sarcopenic muscle is characterized by chronic low-grade inflammation (CLGI), but it is not known if this milieu lowers the inflammasome activation threshold or qualitatively changes inflammasome responses during sepsis. Addressing this question will require longitudinal models that couple aging-, disuse-, or disease-induced sarcopenia with a subsequent septic challenge, enabling investigators to determine whether NLRP3 activation is simply amplified or fundamentally reprogrammed. Complementary spatiotemporal mapping of inflammasome activity across tissues may identify priming effects that static, single-time-point measurements fail to capture.

Another open question is whether restoring mitochondrial quality control can simultaneously improve immune competence and muscle integrity. Mitochondrial dysfunction is a shared hallmark of sepsis and sarcopenia, but it remains unclear whether targeting mitochondrial quality control produces coordinated systemic benefits. Interventions that boost mitophagy and mitochondrial biogenesis, such as modulation of the AMPK/SIRT1/PGC-1α axis, should be evaluated for their dual effects on muscle preservation and immune function. The underlying premise is that restoring mitochondrial homeostasis will limit DAMP release, attenuate inflammatory signaling, and disrupt the feed-forward cycle of immunometabolic dysfunction.

Finally, it remains unclear whether a metabolic tipping point exists beyond which recovery becomes biologically constrained. The transition from adaptive metabolic reprogramming to irreversible energetic failure is poorly defined. Defining thresholds characterized by critical substrate depletion, mitochondrial collapse, or loss of proteostatic control is a major unmet need. Integrating metabolomics, flux analysis, and longitudinal models may help identify these transition points and early biomarkers that distinguish recovery from progression to chronic critical illness or persistent sarcopenia.

In summary, the priority next steps are: (i) causal dissection of muscle-derived signaling (e.g., IL-6, IL-15, PGC-1α) using inducible, muscle-specific loss- and gain-of-function sepsis models; (ii) clarification of how pre-existing sarcopenia reshapes inflammasome activation and inflammatory set points; (iii) systematic evaluation of mitochondrial quality-control restoration as a joint immunometabolic and muscular intervention; (iv) identification of a metabolic tipping point and validation of early biomarkers (e.g., circulating microRNAs, protein isoforms, spatial-transcriptomic signatures) that flag transition to irreversible muscle decline. Addressing these in parallel is essential to move from descriptive association toward targeted, prophylactic intervention.

### 5.1 Levels of evidence of major pathways

The mechanisms discussed above rest on differing levels of evidence, ranging from human clinical data to preclinical models, in vitro studies, and hypothesis-generating concepts. **Table 1** summarizes the major pathways linking sarcopenia and sepsis, indicating the predominant level of evidence.

## 6 Concluding remarks

Future research should progress beyond purely descriptive associations and adopt integrative, systems-level frameworks that can both predict patient trajectories and inform therapeutic decision-making. In this framework, the sepsis-sarcopenia axis may be viewed as a distinct immunometabolic phenotype, arising from the convergence of muscle wasting, mitochondrial dysfunction, and dysregulated inflammatory signaling. By integrating multimodal biomarkers, such as measures of muscle mass and function, mitochondrial performance, and immune activation, clinicians may achieve more accurate patient stratification and identify clinically actionable subtypes. This strategy has the potential to reshape current paradigms by enabling the early recognition of individuals at elevated risk of adverse outcomes, thereby opening a critical window for timely and targeted interventions. Ultimately, transitioning to predictive and personalized models of care may allow disruption of the self-perpetuating cycle of metabolic failure and immune dysfunction before irreversible systemic decline occurs.
